# Effect of Parents’ Encouragement on Reading Motivation: The Mediating Effect of Reading Self-Concept and the Moderating Effect of Gender

**DOI:** 10.3389/fpsyg.2019.00609

**Published:** 2019-03-22

**Authors:** Tiansheng Xia, Honglei Gu, Weirong Li

**Affiliations:** ^1^School of Art and Design, Guangdong University of Technology, Guangzhou, China; ^2^School of Education Science, Xinyang Normal University, Xinyang, China; ^3^College of Foreign Languages, Hunan University, Changsha, China

**Keywords:** parents’ encouragement, reading motivation, reading self-concept, gender, moderated mediation effect

## Abstract

Previous research has found that parental encouragement is associated with children’s motivation to read. However, little is known about the underlying mechanisms of this association or factors that might strengthen or weaken these processes. The current research scrutinized a moderated mediation model that comprised of parental encouragement (predictor variable), reading self-concept (mediator), gender (moderator), and reading motivation (outcome variable) simultaneously. A total of 254 Chinese students (*M*_age_ = 11.35 years, *SD*_age_ = 0.87) completed the Parents’ Encouragement of Extracurricular Reading Questionnaire, Reading Self-Concept Scale, and Pupil Reading Motivation Scale. Path analysis revealed that parents’ encouragement was associated with children’s reading motivation both directly and indirectly via reading self-concept, and the effect of parents’ encouragement on children’s motivation was more positive for boys than girls, while the effect of reading self-concept on children’s motivation was more positive for girls than boys. Our results highlight the importance of parental encouragement in improving children’s reading motivation, and the findings and their implications are discussed.

## Introduction

Reading is a means to understand the external world, the basis for students to learn, and a basic skill for individual survival and development (Smith et al., 2000). However, these benefits depend on the students’ motivation to read. Reading motivation is closely connected to reading comprehension and achievement (Jang et al., 2015; Cartwright et al., 2016) and has been illustrated to forecast the following reading achievement as well (Schaffner et al., 2016). Students with high motivation for reading spend more time on reading activities and show improved ability over time (Schaffner et al., 2013). In contrast, poor readers usually display low motivation to read, and improving reading motivation may be effective in helping poor readers become proficient (Morgan and Fuchs, 2007).

The correlation between reading motivation and reading ability suggests the possibility that each is influenced by the other (Morgan and Fuchs, 2007). That is, reading motivation might promote better reading skills, but better reading skills might also generate more motivation. Other factors might also increase reading motivation. These include disposition, beliefs and goals (Conradi et al., 2014). Disposition refers to positive attitudes about and interest in reading. Beliefs include self-concept about reading (an overall self-perception of oneself as a reader) and self-efficacy about reading (a judgment of one’s capacity of finishing a specific reading task). Goals are an individual’s orientation and intentions toward reading. However, these factors are mostly internal, and situational factors also have an important effect on reading motivation.

Family literacy theory further emphasizes the effects of the learner’s home environment and parent involvement on literacy and achievement. Parents’ encouragement (Shuck et al., 1983) and the home literacy environment (Yeo et al., 2014) are important contextual factors associated with higher reading performance. Parents’ encouragement and praise have also been shown to predict toddlers’ reading comprehension 7 years later (Gunderson et al., 2018). Family literacy theory is also relevant for conceptualizing contextual influences on children’s motivation for reading. For example, previous studies have showed that parent involvement and encouragement have a substantial effect on students’ interest in reading (Baker and Scher, 2002; Yeo et al., 2014). However, the nature of the encouragement is important. The promise of a reward leads to improvement in reading scores in the short term, but encouragement has more lasting effects on student performance (Cheo, 2017).

However, most of these studies have not tested mediators or moderators of the association between parents’ encouragement and children’s reading motivation. Therefore, little is known how these two processes were integrated. In this research, a complicated conceptual model, in which reading self-concept mediated the relation between parents’ encouragement and children’s reading motivation, was tested, and gender moderated these direct and indirect relationships.

### The Mediating Role of Reading Self-Concept

Reading self-concept refers to one person’s whole self-perception as a reader, with one’s sense of competence and the role ascribed to reading as one’s partial personal identity included (Conradi et al., 2014). According to the hypothesis of self-enhancement, self-concept is a fundamental premise for achievement, which may itself be produced by more involvement and effort in reading activities (Marsh and Yeung, 1997). From another perspective, the hypothesis of skill-development implies that academic self-concept results from the achievement, which can be accounted for by social comparisons (Möller and Pohlmann, 2010). For example, two students on the same achievement levels will advance different self-concepts in accordance with the average achievement in their class or school. In addition, self-concept related to reading can affect reading achievement through the mediating effect of reading motivation and meta-cognition (Chapman and Tunmer, 1997), because a positive self-concept is a vital premise for coping with learning difficulties, and in turn, an adaptive response to difficulties encountered in leaning facilitates positive academic process. There may also be reciprocal effects between the self-concept and achievement in reading (Retelsdorf et al., 2014).

Though the individual persons differ in self- and task perceptions and in success expectations, Frome and Eccles (1998) suggested that these differences come directly from children’s interpretation of reality and their parents, but not from the reality itself. Parents’ beliefs and encouragement are important for cultivating their children’s self-concept and competencies, and parents’ reading perception is related to children’s reading self-concept (Frome and Eccles, 1998). Competent parents provide more direct help, encouragement and positive emotional influence in the interaction with their children (Mondell and Tyler, 1981). Such behaviors could directly influence the relationship between parents’ capacity and the child’s reading self-concept and capacity. Grolnick et al. (1991) have ascertained that parents’ behavior does not have direct influence on the children through skill building as traditionally assumed, but through its impact on their attitudes and motivations connected with school. Nevertheless, Bandura et al. (1996) suggested that parents’ encouragement can further develop their children’s academic competence by enhancing their children’s trusts in their academic capacity. The primary indicator of young children’s motivation usually derives from their competency beliefs, and their experiencing early task mastery is supposed to result in higher reading self-concept, and hence greater motivation. To the contrary, declines in motivation is supposed to result from early declines in reading self-concept which would result later in less frequent reading practice (Gottfried, 1990). Harter (1999) reviewed extensive socialization literature on self-concept and concluded that authoritative child rearing is more beneficial than authoritarian or permissive practices. Moreover, social support–especially from parents–is important for forming self-concept. These studies suggest that parents’ self-efficacy and encouragement help shape children’s reading self-concept and academic achievement.

### The Moderating Role of Gender

The gender may influence the reading motivation and reading self-concept. Gender differences in students’ academic self-concept often exceed differences in actual achievement (Hyde and Durik, 2005). Based on the expectancy-value theory, significant others such as parents, peers and teachers would shape the gender stereotypes, which influence the students in many aspects such as their competence beliefs, values, and achievement-related behavior. Gender stereotypes of reading get more support from girls than boys (Retelsdorf et al., 2015).

Generally, girls have higher language-related self-concepts, and boys are believed to have higher mathematics-related self-concepts (Pesu et al., 2016). Indeed, evidence has it that it is reported that girls have higher confidence in their linguistic competence than boys do (Ireson and Hallam, 2009). Moreover, a longitudinal study revealed that these gender differences in language-related self-concepts increase over time across Grade 1–12 (Archambault et al., 2010). A higher proportion of girls maintained strong and stable language-related self-concepts over time; by contrast, a higher proportion of boys indicated substantial decline in language-related self-concepts.

In addition, there appear to be gender differences in reading motivation, reporting that the males have lower motivation for reading than the females (Marinak and Gambrell, 2010; McGeown et al., 2012). Compared to males, females read more frequently (Logan and Johnston, 2009), in both childhood and adolescence. Significant gender differences in reading skills are also frequently reported by children (Martin et al., 2007) and adolescents (Chiu and McBride-Chang, 2006). Compared to girls, other researchers found that reading attitudes, motivation and interest were significantly more strongly related to reading skills for boys (Logan and Johnston, 2009; Logan and Medford, 2011). However, in a recent study of adequate and struggling readers, there was little evidence of consistent gender differences in reading motivation (Wolters et al., 2014).

### The Present Research

**FIGURE 1:**
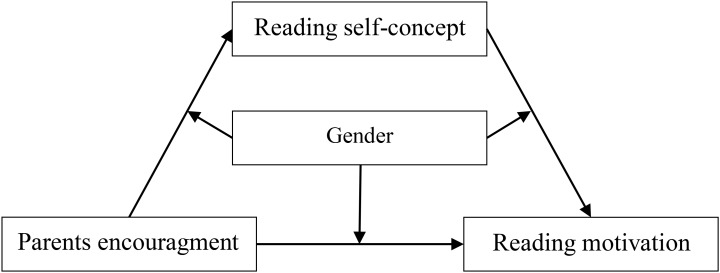
A moderated mediation model predicting reading motivation.

In the present research, parents’ encouragement was designed as the independent variable, reading motivation the dependent variable, reading self-concept the mediating variable, and gender the moderating variable to explore influences on the association between parents’ encouragement and pupils’ reading motivation. We tested the following hypotheses. H1, parents’ encouragement is associated with reading motivation; H2, parents’ encouragement is associated with reading motivation through the mediating effect of reading self-concept; H3, gender moderates the influence of parents’ encouragement on reading motivation. Mediation analysis is a statistical approach used to understand how an independent variable *X* affects a dependent variable *Y* through a mediator *M*, while moderation analysis is used to determine whether the size or sign of the effect of *X* on *Y* depends on (i.e., “interacts with”) a moderator variable (Hayes, 2013). The proposed research model is portrayed in Figure 1.

## Materials and Methods

### Participants

Two hundred and fifty-four students (*M*_age_ = 11.35 years, *SD*_age_ = 0.88) participated in the present study, who were from two primary schools in Zhengzhou, a city located in central China. Among the sample, 18.1% were from Grade 4, 28.0% were from Grade 5, and 53.9% were from Grade 6. Furthermore, 49.2% of these participants were female, and 72.0% had one or more siblings. The majority of participants reported their place of residence as urban (83.9%).

### Measures

#### Parents’ Encouragement

Parents’ encouragement was measured using the Parents’ Encouragement of Extracurricular Reading Questionnaire (PEERQ), a Chinese language measure designed by Gu et al. (2017b). This questionnaire consists of 7 items (e.g., Did your parents give you some reading skills?), and each item was rated on a 5-point Likert scale ranging from 1 (*never*) to 5 (*almost once a day*), and higher scores reflected more encouragement. The PEERQ has shown good reliability and validity in previous research (e.g., Gu et al., 2017a). In the present study, Cronbach’ α for this scale was 0.83. Moreover, confirmatory factor analysis (CFA) suggested that all the factor loadings ranged from 0.56 to 0.78, and the unidimensional model fitted the data well: χ^2^/*df* = 2.35, TLI = 0.96, CFI = 0.97, RMSEA = 0.073, SRMR = 0.031, indicative of good structural validity.

#### Reading Motivation

Reading motivation was measured by 9 items adopted from the Pupil Reading Motivation Scale (PRMS) developed in Chinese by Liu (2012). A sample item from this measure is, “After finishing the reading homework assigned by the teacher, I will do some extra reading exercises by myself.” Each item was rated on a 5-point Likert scale ranging from 1 (*strongly disagree*) to 5 (*strongly agree*). Responses for negative statements were reversely coded, and the average score of all items was calculated so that higher scores reflected higher motivation. In the present study, Cronbach’ α for this measure was 0.76, and CFA showed that all the factor loadings ranged from 0.45 to 0.60, and the one-factor solution fitted the data well: χ^2^/*df* = 2.43, TLI = 0.86, CFI = 0.89, RMSEA = 0.075, SRMR = 0.053.

#### Reading Self-Concept

Reading self-concept was measured by a translated Chinese version of the reading self-concept subscale of the PIRLS Student Questionnaire (Martin et al., 2007), including four items. Each item was rated on a 5-point Likert scale as mentioned above. And the higher scores reflected a more positive self-concept. The reliability and validity has been verified in previous research (e.g., De Naeghel et al., 2012). In the present study, Cronbach’ α for this scale was 0.78, and CFA revealed that all the factor loadings ranged from 0.55 to 0.84, and the unidimensional model fitted the data well: χ^2^/*df* = 3.44, TLI = 0.93, CFI = 0.97, RMSEA = 0.098, SRMR = 0.066.

### Procedure

The assessments were individually administered within a 2-week period in the second month of the academic year, by trained graduate students in a quiet room at the school. Considerable time was taken with these measures to ensure that the response requirements were fully understood and total administration time was around 15 minutes. In order to minimize answering bias (e.g., acquiescence, social desirability), the items of PRMS and RSCS in this study were presented in a randomized order. In addition, we set up a filler item (i.e., I never lie.). If participants answered “strongly agree”, it would be treated as invalid response.

### Data Analysis

In the first place, we employed SPSS software (version 24.0) to analyze descriptive statistics and correlations. In the second, the causal steps approach (Baron and Kenny, 1986) was adopted to investigate the mediation role of reading self-concept in linking parent encouragement and reading motivation. This approach tests the regression coefficients for the effects of predictor on outcome (Step 1), predictor on mediator (Step 2), and mediator on outcome controlling for the predictor (Step 3). Since the causal steps approach does not directly test the mediating effect, and the sampling distribution of mediation effects is often skewed especially for small samples (e.g., *n* < 400), bias-corrected bootstrapping is applied to examine the significance of the mediation effect. As a resampling method, bootstrapping is especially useful when the behavior of a statistic over repeated sampling is either not known, too complicated to derive, or highly context dependent. In mediation analysis, bootstrapping is used to generate an empirically derived representation of the sampling distribution of the indirect effect, and this empirical representation is used for the construction of a confidence interval for αβ (for details, see Hayes, 2013). We can reject the null hypothesis of no mediation if the bootstrapped confidence interval does not contain zero.

Finally, we employing Model 59 of the PROCESS macro to conduct moderated mediation analysis (Step 4 and Step 5) so as to decide whether the indirect path was moderated by gender.

## Results

### Descriptive Statistics

Descriptive statistics and correlations for the measured variables are presented in Table 1. As expected, parent encouragement, reading self-concept, and reading motivation were positively related to each other. Moreover, parent encouragement was negatively associated with place of residence, which indicated that students from urban areas had more parent encouragement than those from rural areas. Age was found to be both negatively associated with parent encouragement and reading motivation, indicating that younger students had more parent encouragement and reading motivation.

**Table 1:** Means, standard deviations, and correlations among the study variables.

Variables	1	2	3	4	5	6	7
1. Age	–						
2. Gender	−0.11	–					
3. Only child	0.04	0.15^∗^	–				
4. Place of residence	0.13^∗^	−0.00	0.11	–			
5. Parent encouragement	−0.19^∗∗^	−0.06	0.02	−0.19^∗∗^	–		
6. Reading self-concept	−0.10	0.08	−0.05	−0.09	0.25^∗∗∗^	–	
7. Reading motivation	−0.14^∗^	0.04	0.02	−−0.02	0.40^∗∗∗^	0.50^∗∗∗^	–
*M*	11.35	0.49	0.72	0.15	3.46	3.94	4.04
*SD*	0.88	0.50	0.45	0.36	1.00	0.84	0.86

*N = 254. Gender was coded as 0, male and 1, female; only child was coded as 0, no and 1, yes; place of residence was coded as 0, rural and 1, urban. ^∗^p < 0.05, ^∗∗^p < 0.01, ^∗∗∗^p < 0.001.*

### Mediation Analyses

Next, the mediation effect of reading self-concept on the association between parent encouragement and reading motivation was tested, and the results were presented in Table 2. After controlling covariates (i.e., age, only child, place of residence, and gender), parent encouragement was significantly correlated with reading motivation (Step 1: β = 0.40, *p <* 0.001) and reading self-concept (Step 2: β = 0.22, *p <* 0.01). Furthermore, when parent encouragement (Step 3: β = 0.30, *p <* 0.001) and reading self-concept (Step 3: β = 0.43, *p <* 0.001) were employed as predictors, they both displayed significant effects on reading motivation. Analysis from bias-corrected bootstrapping with 2000 samples utilizing the PROCESS macro (Hayes, 2013) illustrated a significant mediation effect [*B* = 0.10, *SE* = 0.03, 95%CI (0.04, 0.16)], with parent encouragement still showing a significant direct effect on motivation [*B* = 0.30, *SE* = 0.06, 95%CI (0.19, 0.41)]. The ratio of the mediation effect to the total effect was 0.24 [95%CI (0.11, 0.40)]. Therefore, reading self-concept partially mediated the relation of parent encouragement and reading motivation. Accordingly, both Hypothesis 1 and Hypothesis 2 were supported.

**Table 2:** Tests of the mediation effect and the moderated mediation effect.

	Mediation analyses						Moderated mediation analyses			
	Step 1 (Criterion: RM)		Step 2 (Criterion: RSC)		Step 3 (Criterion: RM)		Step 4 (Criterion: RSC)		Step 5 (Criterion: RM)	
	*b*	*t*	*b*	*t*	*b*	*t*	*b*	*t*	*b*	*t*
Age	−0.06	−0.93	−0.04	−0.55	−0.04	−0.76	−0.04	−0.55	−0.04	−0.78
Only child	0.01	0.10	−0.15	−1.05	0.08	0.64	−0.15	−1.04	0.08	0.71
PR	0.19	1.11	−0.09	−0.50	0.23	1.49	−0.08	−0.47	0.26	1.74
Gender	0.08	0.68	0.16	1.28	0.01	0.11	0.16	1.27	0.01	0.08
PE	0.40	6.49^∗∗∗^	0.22	3.44^∗∗^	0.30	5.38^∗∗∗^	0.25	2.86^∗∗∗^	0.46	6.22^∗∗∗^
RSC					0.43	7.85^∗∗∗^			0.32	4.42^∗∗∗^
PE × Gender							−0.06	−0.48	−0.34	−3.18^∗∗^
RSC × Gender									0.26	2.39^∗^
*R*^2^	0.16		0.07		0.33		0.07		0.37	
*F*	9.29^∗∗∗^		3.33^∗∗^		19.97^∗∗∗^		2.81^∗^		17.30^∗∗∗^	

*N = 254. PR, place of residence; PE, parent encouragement; RSC, reading self-concept; RM, reading motivation. ^∗^p < 0.05, ^∗∗^p < 0.01, ^∗∗∗^p < 0.001.*

### Moderated Mediation Analyses

We employed Model 59 of PROCESS (Hayes, 2013) to investigate whether the mediation effect of reading self-concept was moderated by gender. As seen in Table 2, after controlling covariates (i.e., age, only child, and place of residence), reading self-concept was significantly predicted by parent encouragement (Step 4: β = 0.25, *p* < 0.001), but not by the interaction effect of parent encouragement and gender (Step 4: β = −0.06, *p* > 0.05). The direct effect of reading self-concept on reading motivation was significant (Step 5: β = 0.32, *p* < 0.001), and there was a positive and significant moderation effect of gender between reading self-concept and reading motivation (Step 5: β = 0.26, *p* < 0.05). Moreover, gender was also found to moderate the direct effect of parent encouragement on reading motivation (Step 5: β = –0.34, *p* < 0.01). These observations suggested that both the direct and indirect association between parent encouragement and reading motivation was moderated by gender. More specifically, this was a second stage moderated mediation model, which linked reading self-concept and reading motivation. Thus, Hypotheses 3 was supported.

**FIGURE 2:**
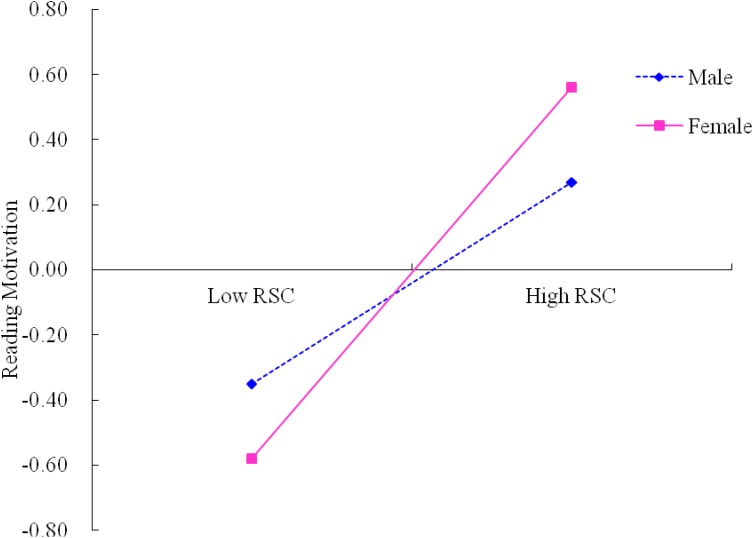
Moderation effect of gender on the relationship between reading self-concept and reading motivation. RSC, reading self-concept.

**FIGURE 3:**
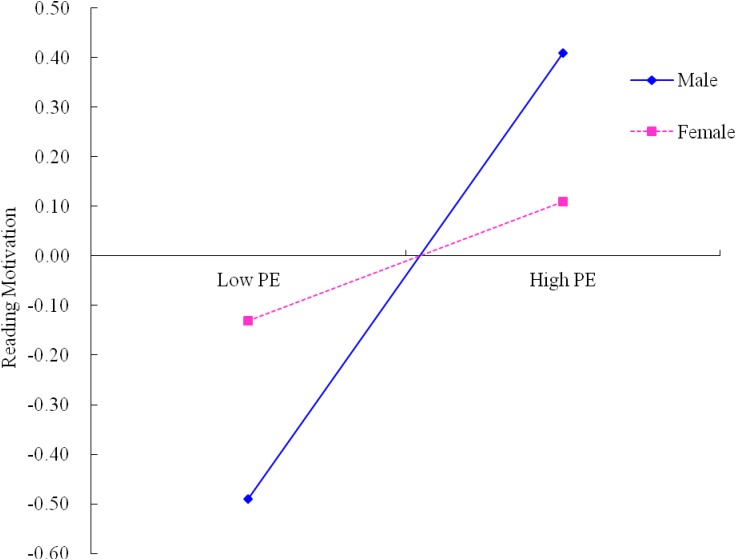
Moderation effect of gender on the relationship between parent encouragement and reading motivation. PE, parent encouragement.

A test of simple slopes was conducted to demonstrate more clearly how gender moderated the influence of parent encouragement and reading self-concept on reading motivation. As revealed in Figure 2, the relation between reading self-concept and reading motivation for females was significant (β_simple_ = 0.58, *p* < 0.001). This relation was way too weaker, however, for males (β_simple_ = 0.32, *p* < 0.001). Furthermore, Figure 3 showed that the direct effect of parent encouragement on reading motivation for males was significant (β_simple_ = 0.46, *p* < 0.001). This effect was much weaker, however, for females (β_simple_ = 0.12, *p* > 0.05). The analysis of conditional indirect effect analysis further illustrated that the whole indirect effect was more noticeable for females [*B* = 0.11, SE = 0.06, 95% CI (0.01, 0.23)], than for males [*B* = 0.08, SE = 0.03, 95% CI (0.03, 0.16)].

## Discussion

The present study examined whether and how parents’ encouragement affects their children’s reading motivation. Parents’ encouragement was directly associated with children’s reading motivation, and this association was partially mediated by children’s reading self-concept. Furthermore, the direct effect of the parents’ encouragement on children’s reading motivation was stronger for boys, and the effect of reading self-concept on children’s reading motivation was stronger for girls.

The direct effect of parents’ encouragement on children’s reading motivation is consistent with Yeo et al. (2014). It is possible that parents who encourage their children to read spend more time interacting with their children in the context of reading, and express more positive emotions about reading activation. This association between positive experiences and reading activity will benefit pupils’ reading motivation.

There was a significant mediating effect of reading self-concept in the association between parents’ encouragement and pupils’ reading motivation, indicating that parents’ encouragement may help to improve pupils’ reading self-concept, which further enriches their reading motivation. Children’s self-concept is mainly derived from their parents, teachers and peers’ beliefs (Pesu et al., 2016). When parents give encouragement, pupils will believe that reading has value and that they have the competency to read. Furthermore, higher reading self-concept usually means that pupils will regard reading as interesting, resulting in higher reading self-efficacy.

Interestingly, the results indicated that the moderating effects of gender between parents’ encouragement and motivation and between reading self-concept and motivation were opposite. Specifically, parents’ encouragement has a stronger impact on boys’ compared to girls’ reading motivation. The possible reason is that girls might be peculiarly vulnerable to the proposed negative effects of personal encouragement resulting in subsequent failure. Traditional socialization practices are inclined to concentrate on dependence and interpersonal relationships for females, while independence and achievement for males (Corpus and Lepper, 2007). Hence, since parents’ encouragement may foreground external evaluation and restrict autonomy for females, it is inclines either to promote feelings of competence or to be neglected by males. Recent study also suggested that parental encouragement and support can more effectively improve boys’ beliefs and motivation than girls’ (Simpkins et al., 2015). In contrast, reading self-concept has a stronger impact on girls’ compared to boys’ reading motivation. The gender stereotype from parents and teachers is that girls outperform boys in reading, and this view has a negative effect on boys’ reading self-concept, but not girls’ (Retelsdorf et al., 2015). Girls persistently demonstrated more positive attitudes toward recreational reading, and greater stability in reading attitudes over time than boys, and they also enjoyed reading significantly more than boys (Marinak and Gambrell, 2010). In addition, they expressed different preferences to the types of books which they read. Specifically, it was reported that boys preferred to read comic and humorous books while girls enjoyed reading adventure books. And even those who had fluent reading abilities weren’t inclined to read aloud (Merisuo-Storm, 2006).

The present results showed that the relation between reading self-concept and reading motivation was stronger for females than males. This moderation effect is likely to be related to gender identity (McGeown et al., 2012). For example, children report that compared to their fathers, their mothers read more, and spend more time teaching them to read (Millard, 1997). Compared with mathematics, science and sports, which are often seen as being more associated with being male, reading is usually regarded as a more feminine activity (Meece et al., 2006). As a result, reading self-concept may affect reading motivation more strongly for girls than for boys.

The findings in the present study suggest that parents’ encouragement can influence their children’s reading motivation directly and through the mediating effect of reading self-concept, raising important implications for students who are eager to improve reading interest and ability. Parents’ encouragement can facilitate the children to stimulate reading motivation and construct higher reading self-concept. As a result, readers in the beginning period who have initial success can indulge in reading for information and pleasure, but lack of parents’ encouragement is bad for the children to develop their reading motivation and reading self-concept. Thus, those who have difficulty and failure in the initial reading period are usually hindered by the less rewarding process of developing basic competence in the lexical level. Once children have stepped into the “swamp” of negative anticipations, lower motivation, and limited practice, they have increasing difficulty in getting back on the road of proficient reading. In addition, the present study suggests that when parents and teachers foster the children’s reading motivation and reading self-concept, they should avoid the negative influence of gender stereotypes on reading self-concept, and give boys more encouragement so as to inspire their reading motivation them through improving their reading self-concepts.

It is necessary for future studies to cope with several limitations innate in the current research. Firstly, though the cross-sectional designs utilized in the present research gives useful information about variable relationships, longitudinal study would be beneficial to verify the causal relationship between parents’ encouragement and children’s reading motivation. Secondly, the collection of our data depended on pupils’ self-report. Though pupils are more susceptible to their own motivation and self-concept than their parents are, at this age social desirability may be a greater influence on girls, whereas boys be tempted to present themselves as less compliant. Multiple informants (e.g., the reports from themselves, their parents, and their teachers) will be beneficial in ruling out the social desirability bias from gender and testing rigorously research hypotheses. Thirdly, this study merely concentrated on the influence of factors in general such as parents’ encouragement and reading self-concept on pupils’ reading motivation. Future research is supposed to further investigate how specific parent’ encouragement, or specific types of books are related to pupils’ reading motivation. Finally, how to turn reading motivation into reading achievement is very important. The main aim in the present study is to investigate the relationship between parental encouragement and child’s reading motivation, but in future research, an objective measure of actual reading achievement would be useful to evaluate the interpretation of our finding, and assess potential reciprocal effects of motivation on reading self-concept.

In summary, this study explored the underlying mechanism of parents’ encouragement on primary school students’ reading motivation. The results suggest some valuable conclusions: (1) parents’ encouragement can influence their children’s reading motivation directly and through the mediating effect of reading self-concept; (2) parents’ encouragement has a stronger impact on boys’ compared to girls’ reading motivation, whereas reading self-concept has a stronger impact on girls’ compared to boys’ reading motivation.

## Data Availability

The datasets for this manuscript are not publicly available. Requests to access the datasets should be directed to guhonglei1985@163.com.

## Ethics Statement

The study protocol was approved by Guangdong University of Technology Research Ethnics Committee, and has been executed consistent with ethical standards laid down in the 1964 Declaration of Helsinki and its later amendments. Written informed consents were obtained from the participants’ parents and all children provided informed assent.

## Author Contributions

TX and HG designed the study and wrote the first draft of the manuscript. HG collected the data, developed, and performed the statistical analysis in conjunction with TX. WL revised critically the final draft together with TX and HG.

## Conflict of Interest Statement

The authors declare that the research was conducted in the absence of any commercial or financial relationships that could be construed as a potential conflict of interest.

## References

[B1] Archambault I. Eccles J. S. Vida M. N. (2010). Ability self-concepts and subjective value in literacy: joint trajectories from grades 1 through 12. *J. Educ. Psychol.* 102 804–816. 10.1037/a0021075

[B2] Baker L. Scher D. (2002). Beginning readers’ motivation for reading in relation to parental beliefs and home reading experiences. *Read. Psychol.* 23 239–269. 10.1080/713775283

[B3] Bandura A. Barbaranelli C. Caprara G. V. Pastorelli C. (1996). Multifaceted impact of self-efficacy beliefs on academic functioning. *Child Dev.* 67 1206–1222. 10.2307/1131888 8706518

[B4] Baron R. M. Kenny D. A. (1986). The moderator–mediator variable distinction in social psychological research: conceptual, strategic, and statistical considerations. *J. Pers. Soc. Psychol.* 51 1173–1182. 10.1037/0022-3514.51.6.1173 3806354

[B5] Cartwright K. B. Marshall T. R. Wray E. (2016). A longitudinal study of the role of reading motivation in primary students’ reading comprehension: implications for a less simple view of reading. *Read. Psychol.* 37 55–91. 10.1080/02702711.2014.991481

[B6] Chapman J. W. Tunmer W. E. (1997). A longitudinal study of beginning reading achievement and reading self-concept. *Br. J. Educ. Psychol.* 67 279–291. 10.1111/j.2044-8279.1997.tb01244.x 9376307

[B7] Cheo R. (2017). Small rewards or some encouragement? Using an experiment in china to test extrinsic motivation on academic performance. *Singapore Econ. Rev.* 62 797–808. 10.1142/S0217590817400276

[B8] Chiu M. M. McBride-Chang C. (2006). Gender, context, and reading: a comparison of students in 43 countries. *Sci. Stud. Read.* 10 331–362. 10.1207/s1532799xssr1004_1

[B9] Conradi K. Jang B. G. McKenna M. C. (2014). Motivation terminology in reading research: a conceptual review. *Educ. Psychol. Rev.* 26 127–164. 10.1007/s10648-013-9245-z

[B10] Corpus J. H. Lepper M. R. (2007). The effects of person versus performance praise on children’s motivation: gender and age as moderating factors. *Educ. Psychol.* 27 487–508. 10.1080/01443410601159852 18816973

[B11] De Naeghel J. Van Keer H. Vansteenkiste M. Rosseel Y. (2012). The relation between elementary students’ recreational and academic reading motivation, reading frequency, engagement, and comprehension: a self-determination theory perspective. *J. Educ. Psychol.* 104 1006–1021. 10.1037/a0027800

[B12] Frome P. M. Eccles J. S. (1998). Parents’ influence on children’s achievement-related perceptions. *J. Pers. Soc. Psychol.* 74 435–452. 10.1037/0022-3514.74.2.435 9491586

[B13] Gottfried A. E. (1990). Academic intrinsic motivation in young elementary school children. *J. Educ. Psychol.* 82 525–538. 10.1037/0022-0663.82.3.525

[B14] Grolnick W. S. Ryan R. M. Deci E. L. (1991). Inner resources for school achievement: motivational mediators of children’s perceptions of their parents. *J. Educ. Psychol.* 83 508–517. 10.1037/0022-0663.83.4.508

[B15] Gu H. Liu J. Lu X. Xia T. (2017a). Effect of parent encouragement on reading autonomy among junior high school students: a mediated moderation model. *Educ. Study Exp.* 6 89–94.

[B16] Gu H. Liu J. Xia T. (2017b). Effect of family socioeconomic status on reading autonomy among elementary school students: the mediating effects of parents’ encouragement and reading motivation. *Acta Psychol. Sin.* 49 1063–1071. 10.3724/SP.J.1041.2017.01063

[B17] Gunderson E. A. Sorhagen N. S. Gripshover S. J. Dweck C. S. Goldin-Meadow S. Levine S. C. (2018). Parent praise to toddlers predicts fourth grade academic achievement via children’s incremental mindsets. *Dev. Psychol.* 54 397–409. 10.1037/dev0000444 29172567 PMC5826820

[B18] Harter S. (1999). *The Construction of the Self: A Developmental Perspective*. New York, NY: Guilford Press.

[B19] Hayes A. F. (2013). *Introduction to Mediation, Moderation, and Conditional Process Analysis: A Regression-Based Approach*. New York, NY: Guilford Press.

[B20] Hyde J. S. Durik A. M. (2005). “Gender, competence, and motivation,” in *Handbook of Competence and Motivation*, eds Elliot A. J. Dweck C. S. Yeager D. S. (New York, NY: Guilford Publications), 375–391.

[B21] Ireson J. Hallam S. (2009). Academic self-concepts in adolescence: relations with achievement and ability grouping in schools. *Learn. Instruct.* 19 201–213. 10.1016/j.learninstruc.2008.04.001

[B22] Jang B. G. Conradi K. McKenna M. C. Jones J. S. (2015). Motivation: approaching an elusive concept through the factors that shape it. *Read. Teach.* 69 239–247. 10.1002/trtr.1365

[B23] Liu Y. (2012). On the development of primary school students’ reading motivations and impacting factors. *Chin. J. Spec. Educ.* 10 90–96.

[B24] Logan S. Johnston R. (2009). Gender differences in reading ability and attitudes: examining where these differences lie. *J. Res. Read.* 32 199–214. 10.1111/j.1467-9817.2008.01389.x

[B25] Logan S. Medford E. (2011). Gender differences in the strength of association between motivation, competency beliefs and reading skill. *Educ. Res.* 53 85–94. 10.1080/00131881.2011.552242

[B26] Marinak B. A. Gambrell L. B. (2010). Reading motivation: exploring the elementary gender gap. *Lit. Res. Instruct.* 49 129–141. 10.1080/19388070902803795

[B27] Marsh H. W. Yeung A. S. (1997). Causal effects of academic self-concept on academic achievement: structural equation models of longitudinal data. *J. Educ. Psychol.* 89 41–54. 10.1037/0022-0663.89.1.41

[B28] Martin M. O. Mullis I. V. Kennedy A. M. (2007). *Progress in International Reading Literacy Study (PIRLS): PIRLS 2006 Technical Report*: ERIC. Boston, MA: TIMSS & PIRLS International Study Center.

[B29] McGeown S. P. Goodwin H. Henderson N. Wright P. (2012). Gender differences in reading motivation: does sex or gender identity provide a better account? *J. Res. Read.* 35 328–336. 10.1111/j.1467-9817.2010.01481.x

[B30] Meece J. L. Glienke B. B. Burg S. (2006). Gender and motivation. *J. School Psychol.* 44 351–373. 10.1016/j.jsp.2006.04.004

[B31] Merisuo-Storm T. (2006). Girls and boys like to read and write different texts. *Scand. J. Educ. Res.* 50 111–125. 10.1080/00313830600576039

[B32] Millard E. (1997). Differently literate: gender identity and the construction of the developing reader. *Gend. Educ.* 9 31–48. 10.1080/09540259721439

[B33] Möller J. Pohlmann B. (2010). Achievement differences and self-concept differences: stronger associations for above or below average students? *Br. .Educ. Psychol.* 80 435–450. 10.1348/000709909X485234 20109275

[B34] Mondell S. Tyler F. B. (1981). Parental competence and styles of problem solving/play behavior with children. *Dev. Psychol.* 17 73–78. 10.1037/0012-1649.17.1.73

[B35] Morgan P. L. Fuchs D. (2007). Is there a bidirectional relationship between children’s reading skills and reading motivation? *Except. Child.* 73 165–183. 10.1177/001440290707300203

[B36] Pesu L. Viljaranta J. Aunola K. (2016). The role of parents’ and teachers’ beliefs in children’s self-concept development. *J. Appl. Dev. Psychol.* 44 63–71. 10.1016/j.appdev.2016.03.001

[B37] Retelsdorf J. Köller O. Möller J. (2014). Reading achievement and reading self-concept–testing the reciprocal effects model. *Learn. Instruct.* 29 21–30. 10.1037/pspp0000230 30667258

[B38] Retelsdorf J. Schwartz K. Asbrock F. (2015). Michael can’t read!” Teachers’ gender stereotypes and boys’ reading self-concept. *J. Educ. Psychol.* 107 186–194. 10.1037/a0037107

[B39] Schaffner E. Philipp M. Schiefele U. (2016). Reciprocal effects between intrinsic reading motivation and reading competence? A cross-lagged panel model for academic track and nonacademic track students. *J. Res. Read.* 39 19–36. 10.1111/1467-9817.12027

[B40] Schaffner E. Schiefele U. Ulferts H. (2013). Reading amount as a mediator of the effects of intrinsic and extrinsic reading motivation on reading comprehension. *Read. Res. Quart.* 48 369–385. 10.1002/rrq.52

[B41] Shuck A. Ulsh F. Platt J. S. (1983). Parents encourage pupils (PEP): an innercity parent involvement reading project. *Read. Teach.* 36 524–528.

[B42] Simpkins S. D. Price C. D. Garcia K. (2015). Parental support and high school students’ motivation in biology, chemistry, and physics: understanding differences among latino and caucasian boys and girls. *J. Res. Sci. Teach.* 52 1386–1407. 10.1002/tea.21246

[B43] Smith M. C. Mikulecky L. Kibby M. W. Dreher M. J. Dole J. A. (2000). What will be the demands of literacy in the workplace in the next millennium? *Read. Res. Quart.* 35 378–383. 10.1598/RRQ.35.3.3

[B44] Wolters C. A. Denton C. A. York M. J. Francis D. J. (2014). Adolescents’ motivation for reading: group differences and relation to standardized achievement. *Read. Writ.* 27 503–533. 10.1007/s11145-013-9454-3

[B45] Yeo L. S. Ong W. W. Ng C. M. (2014). The home literacy environment and preschool children’s reading skills and interest. *Early Educ. Dev.* 25 791–814. 10.1080/10409289.2014.862147

